# Circadian Rhythm Genes *CLOCK* and *PER3* Polymorphisms and Morning Gastric Motility in Humans

**DOI:** 10.1371/journal.pone.0120009

**Published:** 2015-03-16

**Authors:** Mitsue Yamaguchi, Kazuhiko Kotani, Kokoro Tsuzaki, Ayaka Takagi, Naoko Motokubota, Naho Komai, Naoki Sakane, Toshio Moritani, Narumi Nagai

**Affiliations:** 1 Graduate School of Human Science and Environment, University of Hyogo, Hyogo, Japan; 2 Department of Clinical Laboratory Medicine, Jichi Medical University, Shimotsuke-Tochigi, Japan; 3 Division of Preventive Medicine, Clinical Research Institute for Endocrine and Metabolic Disease, National Hospital Organization, Kyoto Medical Center, Kyoto, Japan; 4 Laboratory of Applied Physiology, Graduate School of Human and Environmental Studies, Kyoto University, Kyoto, Japan; Pennsylvania State University, UNITED STATES

## Abstract

**Background:**

Clock genes regulate circadian rhythm and are involved in various physiological processes, including digestion. We therefore investigated the association between the *CLOCK* 3111T/C single nucleotide polymorphism and the *Period3* (*PER3*) variable-number tandem-repeat polymorphism (either 4 or 5 repeats 54 nt in length) with morning gastric motility.

**Methods:**

Lifestyle questionnaires and anthropometric measurements were performed with 173 female volunteers (mean age, 19.4 years). Gastric motility, evaluated by electrogastrography (EGG), blood pressure, and heart rate levels were measured at 8:30 a.m. after an overnight fast. For gastric motility, the spectral powers (% normal power) and dominant frequency (DF, peak of the power spectrum) of the EGG were evaluated. The *CLOCK* and *PER3* polymorphisms were determined by polymerase chain reaction (PCR) restriction fragment length polymorphism analysis.

**Results:**

Subjects with the *CLOCK* C allele (T/C or C/C genotypes: n = 59) showed a significantly lower DF (mean, 2.56 cpm) than those with the T/T genotype (n = 114, 2.81 cpm, *P* < 0.05). Subjects with the longer *PER3* allele (*PER3* ^4/5^ or *PER3* ^5/5^ genotypes: n = 65) also showed a significantly lower DF (2.55 cpm) than those with the shorter *PER3* ^4/4^ genotype (n = 108, 2.83 cpm, *P* < 0.05). Furthermore, subjects with both the T/C or C/C and *PER3* ^4/5^ or *PER3* ^5/5^ genotypes showed a significantly lower DF (2.43 cpm, *P* < 0.05) than subjects with other combinations of the alleles (T/T and *PER3* ^4/4^ genotype, T/C or C/C and *PER3* ^4/4^ genotypes, and T/T and *PER3* ^4/5^ or *PER3* ^5/5^ genotypes).

**Conclusions:**

These results suggest that minor polymorphisms of the circadian rhythm genes *CLOCK* and *PER3* may be associated with poor morning gastric motility, and may have a combinatorial effect. The present findings may offer a new viewpoint on the role of circadian rhythm genes on the peripheral circadian systems, including the time-keeping function of the gut.

## Introduction

The endogenous circadian clock regulates daily oscillations in various behavioral and physiological processes of the human body, including those associated with digestive activity [1]. In mammals, the core molecular clock is present within the hypothalamic suprachiasmatic nucleus (SCN) in the brain, and in nearly all peripheral tissues [1, 2]. The peripheral clocks are organ-specific and tend to be delayed by 3–9 hours when compared to the SCN [3]; therefore, the SCN neurons synchronize peripheral tissue clocks through both neuronal and hormonal pathways [4, 5].

The SCN and peripheral core molecular clocks consist of interconnected positive and negative transcription and translation feedback loops that oscillate every 24 hours [1, 2, 4]. In this system, the forward loop involves a set of transcriptional enhancers that induce the transcription of a set of repressors, and the negative loop feeds back to inhibit the forward loop [3–5]. The core clock genes (*CLOCK*, *Bmal1*, *Period*, *Cry*) are considered essential elements of this biological molecular system, and genetic variants such as the *CLOCK* 3111T/C single nucleotide polymorphism (SNP) or the *Period3* (*PER3*) variable-number tandem-repeat (VNTR) polymorphism (either 4 or 5 repeats 54 nt in length) may contribute to the disruption of the circadian clock [6–8].

Peripheral tissue clocks also exist in the gastrointestinal tract (GIT), located in special intestinal cells, with unstable membrane potentials positioned between the longitudinal and circular muscle layers [9–12]. These gut clocks are responsible for the periodic activity of various segments and transit along the GIT [9]. The migrating motor complex starts in the stomach and moves along the gut causing peristaltic contractions when the electrical activity spikes are superimposed on the slow waves [9]. The rhythm of slow waves occurs in the stomach (3 cycles per minute [cpm]), duodenum (12 cpm), jejunum and ileum (7–10 cpm), and colon (12 cpm), and can be cooperatively controlled by the gut and brain SCN clocks [9].

With respect to the stomach, gastric motility shows circadian rhythm as well as mealtime variations in healthy subjects [13]. Similarly, it has been reported that ghrelin, an appetite regulating hormone produced by the stomach, is secreted in anticipation of regularly scheduled mealtime [14]. Indeed, the expression of ghrelin and *PER* is rhythmic in light-dark cycles and is synchronized to prior feeding, suggesting that the anticipatory activity, such as gastric motility before breakfast, depends on an endogenous circadian timing system [14]. This system called circadian food-entrainable oscillators (FEOs) is closely related to both brain and peripheral clocks, and is located in oxyntic gland cells of stomach [9, 14]. In healthy subjects, ghrelin concentration elevated 1–2 h before breakfast [15], and an increase in gastric motility occurs several hours before the meal [9]. These findings raise the possibility that attenuated gastric locomotor activity before breakfast may reflect the modulation of peripheral and/or central circadian timing system.

Accordingly, we have previously demonstrated the involvement of *CLOCK* on gut function; subjects with the *CLOCK* 3111T/C SNP showed slower gastric waves (mean, 2.45 cpm) compared to those with the T/T genotype (2.94 cpm) [16]. Given the existence of several circadian clock genes [6–8], the impact of other clock gene polymorphisms on gut function should further be investigated. We hypothesized that the *CLOCK* 3111T/C SNP and *PER3* VNTR polymorphism may modulate gastric contraction rhythm. Therefore, the present study was designed to investigate sole and combinatorial involvement of these polymorphisms with morning gastric motility in healthy female volunteers.

## Materials and Methods

### Subjects

We recruited 173 female university students (19.4 ± 0.1 years) from our campus for the present study. All subjects were non-smokers, free of any symptoms or medical history of gastrointestinal, cardiovascular, or any diseases that affect gastric motility, and were not taking any medications. The study protocol was reviewed and approved by the Ethics Committee of the University of Hyogo, and was in accordance with the principles of the Declaration of Helsinki. All subjects provided written informed consent.

### Experimental protocol

All subjects were asked to maintain their usual lifestyle and body weight for at least one month before the test. On the day before the test, the consumption of coffee, tea, spicy foods, and high-fat foods was prohibited. Subjects were tested in the morning, from 8:00 to 9:00 a.m., after an overnight fast beginning at 10:00 p.m. of the previous night. After measurement of body mass and percent body fat were using a bioelectrical impedance analyzer (InBody520, Biospace Co., Seoul, Korea), seated blood pressure levels were measured twice at 5 min intervals using a sphygmomanometer (HEM-7250-IT; OMRON, Co., Ltd., Kyoto, Japan). Subjects were then prepared for electrogastrography (EGG) and electrocardiogram (ECG) and allowed to rest for at least 15 min in a temperature-controlled (24–25°C) room in a sitting-up, 45° inclined position. After the resting period, EGG and ECG were continuously recorded for 20 min. The resting heart rate (HR) values were obtained from ECG data. The subjects were separated by partition screens and instructed to maintain their positions for the duration of data collection.

### EGG measurement and spectral analysis procedure

To derive bipolar EGG signals, two active electrodes and one ground were positioned on the abdomen, as suggested by the American Motility Society Clinical GI Motility Testing Task Force [17]. The high cut-off frequency of the EGG amplifier was 0.15 Hz, and the low cut-off frequency was 0.016 Hz. These band-pass filters completely eliminated ECG and 60 Hz power source artifacts [16, 18]. The EGG signals were amplified (EGG Amplifier BBA-8321, Bio-tex, Kyoto, Japan) and digitized via a 13-bit analog-to-digital converter (DAQ AD135, Germany) at a sampling rate of 0.5 Hz. The acquired data were sequentially stored on a hard disk for later analysis. The root mean square value of the EGG was calculated as representing the average amplitude. After passing through the hamming-type data window, power spectral analysis by means of a fast Fourier transformation was performed on a consecutive 512 time point series of data obtained during the test. Signal acquisition, storage, and processing were performed on a personal computer. The computer programs for sampling and analysis were written in HTBasic (Trans Era version 9.0, Utah, USA).

**Fig 1:**
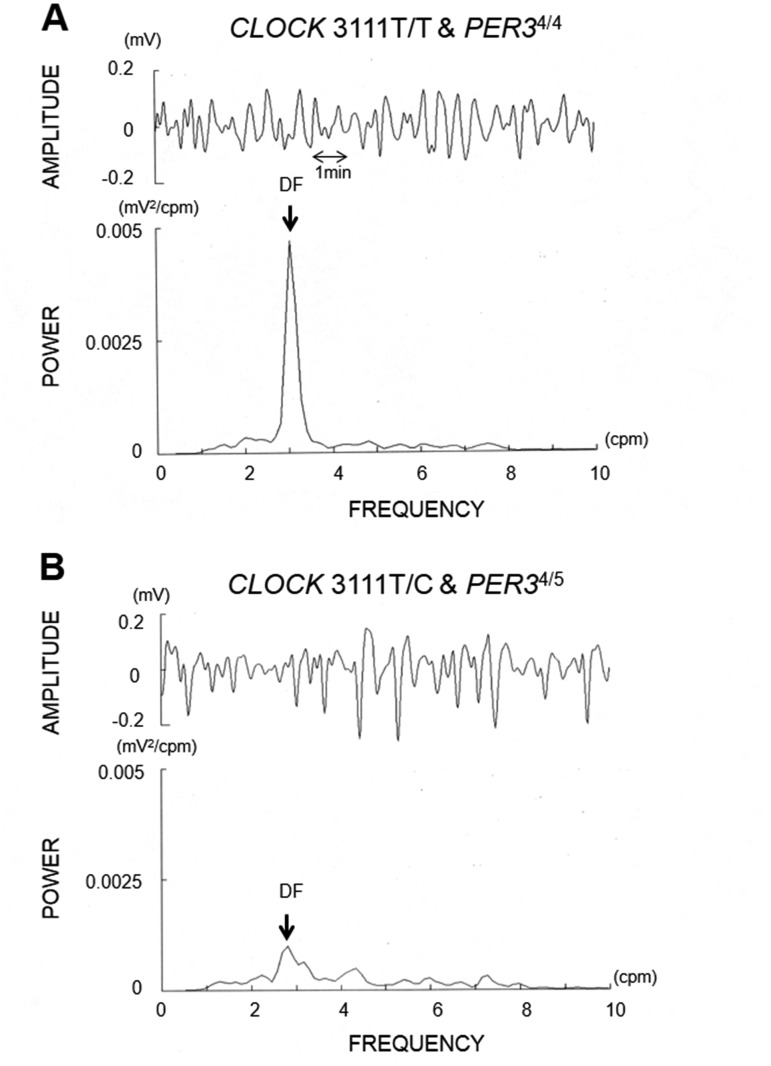
EGG power spectrum analysis results. Typical sets of raw EGG waves (top of each figure) and the corresponding power spectral data (bottom of each figure) obtained from a T/T and *PER3* ^4/4^ (A) and a T/C and *PER3* ^4/5^ genotype subject (B). By visual inspection, in the T/C and *PER3* ^4/5^ genotype subject, the DF of the waves was diminished and shifted toward the slower frequency compared to the T/T and *PER3* ^4/4^ genotype subject.

To determine gastric motility, the components of gastric slow wave, normal power (2–4 cpm), % normal power (normal power / total power derived from 1–9 cpm), and dominant frequency (DF) were calculated [16–18]. Raw gastric myoelectrical signals (amplitude) and the corresponding power spectrum are illustrated in Fig. 1.

### Dietary intake and breakfast frequency

Dietary intake and nutritional values were estimated from self-reported, weighed food records with photographs of all consumed food and drink taken using a camera-equipped cellular phone for two typical weekdays. These records were carefully checked by registered dietitians through an interview with each subject on the test day [16, 18]. Energy intake and nutritional values were calculated using computer-assisted procedures (Excel Eiyokun, ver. 6.0, Kenpakusya Co., Tokyo, Japan) based on the Japanese food composition table. A lifestyle questionnaire was also performed; breakfast frequency was divided into three categories to make clear comparisons among subjects who consumed breakfast daily, intermittently, or never.

### Genetic analysis

DNA samples were obtained from whole blood using a DNA Quick II, genomic DNA separation kit (DS Pharma Biomedical Co., Ltd., Osaka, Japan). The polymorphisms of *CLOCK* and *PER3* were determined by a polymerase chain reaction (PCR) restriction fragment length polymorphism analysis. The 3111T/C polymorphic region of *CLOCK* (rs1801260) was amplified using PCR with forward (5’- TCC AGC AGT TTC ATG AGA TGC-3’) and reverse (5’-GAG GTC ATT TCA TAG CTG AGC-3’) primers. The VNTR polymorphic region of *PER3* (rs57875989) was amplified using PCR with forward (5'-TTC TAG CAG TGT GTT ACA GGC AAC-3') and reverse (5'-TCC TGA TGC TGC TGA ACC AGT TC-3') primers. PCR amplification was performed under the conditions recommended by the enzyme supplier. In brief, a 10-μL reaction contained 200 ng of genomic DNA, the reaction buffer supplied, 3.0 mM MgCl_2_, 0.2 mM dNTP, and 1.25 U of rTaq (TAKARA Bio Inc., Tokyo, Japan). For the 3111T/C polymorphic region of *CLOCK*, cycling parameters were an initial denaturation at 94°C for 3 minutes, then denaturation at 95°C for 30 seconds, annealing at 58°C for 30 seconds, and primer extension at 72°C for 60 seconds for five cycles, then denaturation at 95°C for 30 seconds, annealing at 55°C for 30 seconds, and primer extension at 72°C for 60 seconds for thirty cycles in a thermal cycler (GeneAmp PCR System 2700). The 3111T/C polymorphism was identified by restriction of the 221bp PCR-fragment with *Bsp1286I*; the C-allele is cut by *Bsp1286I*. In the VNTR polymorphic region of *PER3*, cycling parameters were an initial denaturation at 94°C for 3 minutes, then denaturation at 94°C for 30 seconds, annealing at 65°C for 30 seconds, and primer extension at 72°C for 30 seconds for thirty-five cycles, and post-extension at 72°C for 4 minutes in a thermal cycler. The resultant PCR products (335bp and/or 282bp) were separated on 1.5% agarose gels and visualized using ethidium bromide. All samples were checked by two independent investigators, and no samples showed differing results between the investigators.

### Statistical analyses

The data were expressed as the mean ± standard errors (s.e.). Comparison between two groups (T/T *vs*. T/C or C/C genotypes, *PER3* ^4/4^ *vs*. *PER3* ^4/5^ or *PER3* ^5/5^ genotypes) was tested by unpaired Student’s *t*-test. Comparison among four groups (T/T and *PER3* ^4/4^ genotype, T/C or C/C and *PER3* ^4/4^ genotypes, T/T and *PER3* ^4/5^ or *PER3* ^5/5^ genotypes, and T/C or C/C and *PER3* ^4/5^ or *PER3* ^5/5^ genotypes) was tested by one way analysis of variance (ANOVA) using post hoc Tukey’s test. In ANOVA, we performed adjustments of the association by multiple regression analysis, adjusted for age, body mass index (BMI) and the four genotype groups. Comparison of breakfast frequency between genotype groups was analyzed by χ^2^-tests or Fisher exact tests, as appropriate. All analyses were performed using SPSS 20 for Windows (IBM Inc., Tokyo, Japan). *P* < 0.05 was considered significant.

## Results

### Distribution of genotypes

Table 1 shows subject characteristics by *CLOCK* and *PER3* polymorphisms. The frequency of the *CLOCK* −3111 C allele was 0.18, and the longer *PER3* allele was 0.21. The genotype frequencies of *CLOCK* T/T, T/C, and C/C were 0.66, 0.32, and 0.02, and *PER3* ^4/4^, *PER3* ^4/5^, and *PER3* ^5/5^ were 0.63, 0.32, and 0.05, respectively. These genotype frequencies were in Hardy-Weinberg equilibrium (*P* > 0.05). The *CLOCK* −3111 T/C and longer *PER3* polymorphisms were independent of one other in terms of linkage disequilibrium.

**Table 1:** Subject characteristics according to genotype of *CLOCK* and *PER3*.

		Genotype					
		*CLOCK* 3111T/C			*PER3* VNTR		
	Total	T/T	T/C or C/C	*P* value^a^	4/4	4/5 or 5/5	*P* value^a^
Number	173	114	59	–	108	65	–
Age (years)	19.4 ± 0.1	19.6 ± 0.2	18.9 ± 0.1	0.015	19.3 ± 0.2	19.5 ± 0.2	0.41
Height (cm)	158.5 ± 0.4	158.4 ± 0.5	158.7 ± 0.7	0.73	159.0 ± 0.5	157.8 ± 0.6	0.12
Body mass (kg)	51.0 ± 0.5	50.3 ± 0.5	52.3 ± 0.9	0.042	51.3 ± 0.7	50.5 ± 0.7	0.42
Body mass Index (kg/m^2^)	20.3 ± 0.2	20.0 ± 0.2	20.7 ± 0.3	0.046	20.3 ± 0.2	20.3 ± 0.2	1.00
Body fat (%)	26.1 ± 0.4	25.7 ± 0.5	26.8 ± 0.7	0.21	25.9 ± 0.5	26.5 ± 0.7	0.50
Systolic blood pressure (mmHg)	98.3 ± 0.6	98.2 ± 0.7	98.3 ± 0.9	0.92	98.3 ± 0.7	98.1 ± 0.9	0.87
Diastolic blood pressure (mmHg)	59.3 ± 0.5	60.0 ± 0.7	58.0 ± 0.8	0.067	59.9 ± 0.7	58.5 ± 0.8	0.19
Heart rate (bpm)	65.6 ± 0.7	67.1 ± 0.8	62.8 ± 1.0	0.002	65.9 ± 0.9	65.0 ± 0.9	0.53
Body temperature (°C)	36.11 ± 0.32	36.13 ± 0.04	36.08 ± 0.06	0.54	36.11 ± 0.04	36.12 ± 0.06	0.88
% normal power (%)	50.3 ± 1.3	51.6 ± 1.5	47.8 ± 2.2	0.15	51.1 ± 1.5	48.9 ± 2.2	0.39
Dominant frequency (cpm)	2.72 ± 0.06	2.81 ± 0.07	2.56 ± 0.12	0.049	2.83 ± 0.07	2.55 ± 0.11	0.021
Enegy intake (kJ/day)	6735 ± 122	6611 ± 146	6987 ± 209	0.14	6669 ± 163	6853 ± 172	0.47
Sleep duration (hour)	6.08 ± 0.76	6.06 ± 0.10	6.12 ± 0.13	0.68	6.11 ± 0.10	6.03 ± 0.11	0.66
Breakfast frequency							
Daily	141 (81.5)	91 (79.8)	50 (84.7)		89 (82.4)	52 (80.0)	
Intermettently	26 (15.0)	18 (15.8)	8 (13.6)	0.59^b^	14 (13.0)	12 (18.5)	0.38^b^
Never	6 (3.5)	5 (4.4)	1 (1.7)		5 (4.6)	1 (1.5)	

Values are expressed as subject numbers or mean ± s.e. or number and frequency (%).

^a^ Unpaired Student's *t*-test (T/T *vs*. T/C or C/C, *PER3* ^4/4^ *vs*. *PER3* ^4/5 or 5/5^).

^b^ χ^2^-tests or Fisher's exact test (T/T *vs*. T/C or C/C, *PER3* ^4/4^ *vs*. *PER3* ^4/5 or 5/5^).

### The association of *CLOCK* or *PER3* polymorphisms with biological parameters

To examine the association between the *CLOCK* C allele and biological parameters, subjects were divided into two groups either with (T/C or C/C genotypes) or without (T/T genotype) the C allele. Likewise, to examine the association between the longer *PER3* allele and biological function, subjects were divided into two groups either with (*PER3* ^4/5^ or *PER3* ^5/5^ genotypes) or without (*PER3* ^4/4^ genotype) the longer allele. Carriers of the *CLOCK* C allele showed significantly higher body mass and BMI compared to subjects with the T/T genotype. Additionally, resting HR and the DF were significantly lower in C allele carriers than in T/T genotype subjects, in confirmation with our previous report [16]. Carriers of the longer *PER3* allele (*PER3* ^4/5^ or *PER3* ^5/5^ genotypes) had a significantly lower DF compared with subjects with the *PER3* ^4/4^ genotype. No significant differences were found in breakfast frequency and other parameters between the groups (Table 1).

### Combinatorial effect of *CLOCK* and *PER3* polymorphisms on biological parameters

Subjects were classified into four combination groups of genotypes based on two gene polymorphisms; T/T and *PER3* ^4/4^ genotype (frequency, 0.42), T/C or C/C and *PER3* ^4/4^ genotypes (0.20), T/T and *PER3* ^4/5^ or *PER3* ^5/5^ genotypes (0.24), and T/C or C/C and *PER3* ^4/5^ or *PER3* ^5/5^ genotypes (0.14), respectively, to examine the combinatorial effect of each allele on biological parameters (Table 2).

**Table 2:** Comparison of biological parameters and breakfast frequency among four genotype groups.

		Genotype				
	Total	T/T	T/C or C/C	T/T	T/C or C/C	*P* value^a^
		4/4	4/4	4/5 or 5/5	4/5 or 5/5	
Number	173	73	35	41	24	–
Age (years)	19.4 ± 0.1	19.5 ± 0.2	18.7 ± 0.2	19.7 ± 0.3	19.2 ± 0.2	0.065
Height (cm)	158.5 ± 0.4	158.7 ± 0.6	159.7 ± 1.0	158.1 ± 0.8	157.3 ± 0.9	0.29
Body mass (kg)	51.0 ± 0.5	50.1 ± 0.7	53.7 ± 1.3^b^	50.6 ± 0.9	50.4 ± 1.1	0.061
Body mass Index (kg/m^2^)	20.3 ± 0.2	19.9 ± 0.3	21.0 ± 0.4	20.2 ± 0.3	20.3 ± 0.4	0.11
Body fat (%)	26.1 ± 0.4	25.3 ± 0.6	27.1 ± 1.0	26.5 ± 0.9	26.4 ± 1.0	0.39
Systolic blood pressure (mmHg)	98.3 ± 0.6	98.3 ± 1.0	98.3 ± 1.0	98.0 ± 1.1	98.4 ± 1.8	1.00
Diastolic blood pressure (mmHg)	59.3 ± 0.5	60.6 ± 0.9	58.3 ± 1.0	59.0 ± 1.0	57.6 ± 1.3	0.17
Heart rate (bpm)	65.6 ± 0.7	67.1 ± 1.2	63.4 ± 1.3	67.0 ± 1.1	61.8 ± 1.5^b^	0.017
Body temperature (°C)	36.11 ± 0.32	36.12 ± 0.05	36.09 ± 0.07	36.14 ± 0.07	36.07 ± 0.11	0.92
% normal power (%)	50.3 ± 1.3	53.7 ± 1.7	45.6 ± 2.9	47.7 ± 2.8	50.9 ± 3.3	0.069
Dominant frequency (cpm)	2.72 ± 0.06	2.92 ± 0.07	2.65 ± 0.15	2.62 ± 0.13	2.43 ± 0.19^b^	0.030
Enegy intake (kJ/day)	6735 ± 122	6514 ± 200	7073 ± 300	6764 ± 209	6887 ± 280	0.37
Sleep duration (hour)	6.08 ± 0.76	6.06 ± 0.13	6.19 ± 0.17	6.04 ± 0.14	6.03 ± 0.20	0.91
Breakfast frequency						
Daily	141 (81.5)	60 (82.2)	29 (82.9)	31 (75.6)	21 (87.5)	
Intermettently	26 (15.0)	9 (12.3)	5 (14.3)	9 (22.0)	3 (12.5)	0.69^c^
Never	6 (3.5)	4 (5.5)	1 (2.8)	1 (2.4)	0 (0)	

Values are expressed as subject numbers or mean ± s.e. or number and frequency (%).

^a^ ANOVA using post hoc Turkey's test.

^b^ *P* < 0.05 *vs*. T/T and *PER3* ^4/4^

^c^ χ^2^-tests or Fisher's exact test.

#### Blood pressure

Average systolic and diastolic BP of the four groups was shown in Table 2. There was no significant difference among the four groups.

#### Resting heart rate

**Fig 2:**
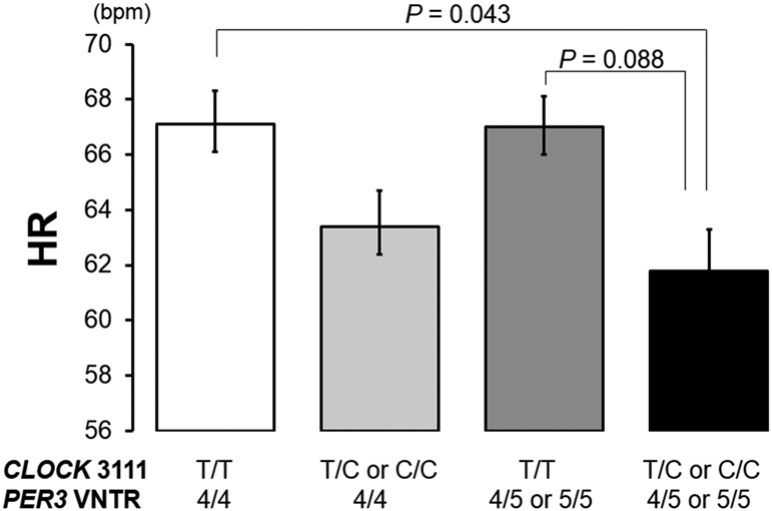
The resting HR of the four combined polymorphic types. Subjects with both T/C or C/C and *PER3* ^4/5^ or *PER3* ^5/5^ genotypes displayed the lowest resting HR values among the four groups. Additionally, there was a significant difference in the resting HR between the T/T and *PER3* ^4/4^ genotype and T/C or C/C and *PER3* ^4/5^ or *PER3* ^5/5^ genotypes (ANOVA followed by post-hoc Tukey’s test).

Average resting HR of the four groups was shown in Table 2. There were significant differences in the resting HR among the four groups (ANOVA, *F* = 3.49, *P* = 0.017). As shown in Fig. 2, subjects with the T/C or C/C and *PER3* ^4/5^ or *PER3* ^5/5^ genotypes had a significantly lower resting HR compared with subjects with the T/T and *PER3* ^4/4^ genotype (*P* = 0.043), indicating a combinatorial effect of the *CLOCK* −3111 C and longer *PER3* alleles on decreased resting HR in the morning. Additionally, the resting HR of subjects with the T/C or C/C and *PER3* ^4/5^ or *PER3* ^5/5^ genotypes tended to be lower than in subjects with T/T and *PER3* ^4/5^ or *PER3* ^5/5^ genotypes (*P* = 0.088). After adjustment for age (*β* = 0.09, *P* = 0.26) and BMI (*β* = −0.05, *P* = 0.48), the difference among four genotype groups remained significant (*R* = 0.25, *β* = −0.21, *P* = 0.007).

#### EGG parameters

Average % normal power of the four groups was shown in Table 2. The % normal power of subjects with the T/T and *PER3* ^4/4^ genotype tended to be greater than in subjects with the other genotypes (ANOVA, *F* = 2.41, *P* = 0.069).

#### DF

**Fig 3:**
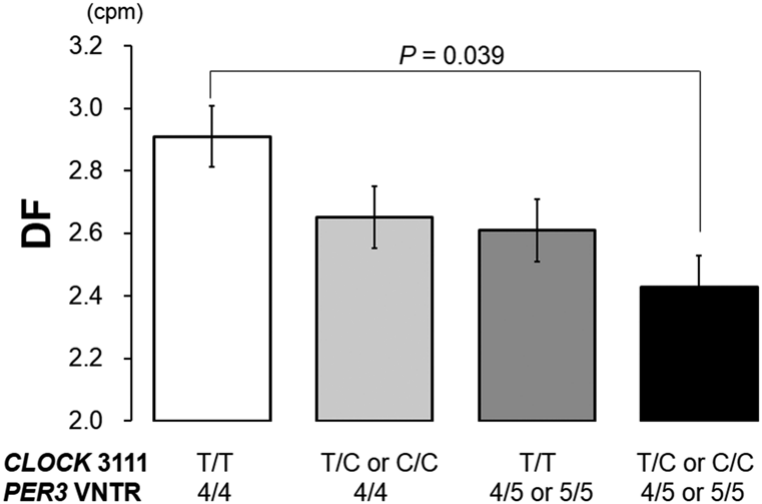
The DF of the four combined polymorphic types. Subjects with both T/C or C/C and *PER3* ^4/5^ or *PER3* ^5/5^ genotypes displayed the lowest DF values among the four groups, suggesting the slowest gastric motility. Additionally, there was a significant difference in the DF between the T/T and *PER3* ^4/4^ genotype and T/C or C/C and *PER3* ^4/5^ or *PER3* ^5/5^ genotypes (ANOVA followed by post-hoc Tukey’s test).

Fig. 1 represents typical sets of raw EGG waves (top of each figure) and the corresponding power spectral data (bottom of each figure) obtained from a T/T and *PER3* ^4/4^ (A) and a T/C and *PER3* ^4/5^ (B) subject. By visual inspection, in the T/C and *PER3* ^4/5^ subject, the DF of the waves was diminished and shifted toward the slower frequency compared to the T/T and *PER3* ^4/4^ subject. The group data indicated that there were significant differences in the DF among the four groups (ANOVA, *F* = 3.06, *P* = 0.030) (Table 2); combination of the *CLOCK* −3111 C and longer *PER3* alleles had a stronger impact on the DF than that of either polymorphism. As shown in Fig. 3, subjects with the T/C or C/C and *PER3* ^4/5^ or *PER3* ^5/5^ genotypes displayed the lowest DF values among the four groups, suggesting the slowest gastric motility. Additionally, a significant difference in the DF was found between the T/T and *PER3* ^4/4^ genotype and the T/C or C/C and *PER3* ^4/5^ or *PER3* ^5/5^ genotypes (*P* = 0.039) (Fig. 3). After adjustment for age (*β* = 0.07, *P* = 0.35) and BMI (*β* = –0.05, *P* = 0.51), the difference among four genotype groups remained significant (*R* = 0.23, *β* = –0.19, *P* = 0.013). These results imply a combinatorial effect of the *CLOCK* −3111 C and longer *PER3* alleles on morning gastric motility in the fasting condition.

No significant differences were found among the four groups for energy intake, breakfast frequency, or sleep duration (Table 2). These variables did not have any association with the measured biological parameters (data not shown).

## Discussion

Genetic and metabolic circadian oscillators are coupled to synchronize between the light-dark/feeding-fasting cycles and human internal biochemical processes [19]. Therefore, genetic variations in the circadian rhythm genes may influence not only circadian time-keeping but also the energetic cycles in humans [19]. The present study examined the association of the *CLOCK* 3111T/C SNP and *PER3* VNTR polymorphism with morning gastric motility in humans. The novelty of this study is that 1) the subjects carrying the minor allele at *CLOCK* or *PER3* possessed significantly lower DF values, suggesting poor gastric motility compared to non-carrier subjects, and that 2) the subjects carrying both minor alleles at *CLOCK* and *PER3* showed the lowest DF value of the groups, suggesting this combined polymorphism may be linked to a greater suppression of gastric motility in the morning.

It has been thought that the *CLOCK* 3111T/C SNP is associated with higher plasma ghrelin concentrations, altered eating behaviors, higher total energy intake, and resistance to weight loss [20–22], implying a genetic susceptibility to obesity [7]. Our subjects with the *CLOCK* C allele also had a modest but significantly higher BMI than subjects with the T/T genotype. Interestingly, a recent report [23] using actimetry observed that *CLOCK* C allele carriers were less active and started their activities later in the morning. In the present study, *CLOCK* C allele carriers showed decreased HR, slightly lower diastolic BP, and poor gastric motility in the morning. Such attenuated cardiovascular function together with the gastric function that characterizes C allele carriers may be attributable to behavior or performance after wake-up.

Intriguingly, not only sole (*CLOCK*) but combinatorial effect of *CLOCK* and *PER3* VNTR miner alleles on the resting HR were observed in the present study. In an animal study [24], cardiomyocyte-specific circadian clock mutant mice showed bradycardia and attenuation of HR diurnal variations, suggesting that peripheral *CLOCK* gene in heart may directly influence myocardial contractile function. A grovel understanding of the role of circadian clock genes in cardiovascular function is still lacking in humans; however, there are a few reports regarding the association between *CLOCK* or *PER3* polymorphism and the resting HR. As to the *CLOCK* 3111T/C SNP, we recently reported that the *CLOCK* C allele carriers had a lower resting HR in the morning [16]. In agreement of the present results, a previous research suggested that the *PER3* VNTR polymorphism was not associated with resting HR at any time point [25]. By interacting with *CLOCK*; however, *PER3* could have a greater impact on cardiovascular function or parasympathetic-sympathetic nervous system balance in the morning.

The role of *PER3* in regulating metabolism and adiposity has been described in animal studies [26, 27], but is not yet clear in humans [28]. We found no relationship between the *PER3* polymorphism and BMI, suggesting this polymorphism may have only a relatively modest effect on metabolic function. On the other hand, previous findings regarding the role of *PER3* on habitual and behavioral circadian rhythm in humans have been controversial. The shorter 4-repeat [29–31] or longer 5-repeat [32] alleles of *PER3* have been associated with diurnal preference. Earlier wake/bed time [33] and favorable sleep homeostasis [34, 35] were observed in the longer 5-repeat allele carriers. However, some studies reported no correlation between the *PER3* polymorphism and habitual sleep patterns or morningness-eveningness phenotypes in healthy volunteers [34–37] and university students [38].

In the present study, although usual sleep duration as well as breakfast frequency did not differ across genotype groups, the longer 5-repeat allele (*PER3* ^4/5^ or *PER3* ^5/5^ genotypes) showed slower morning gastric motility. Interestingly, recent investigations have emphasized that the *PER3* ^5/5^ genotype has a detrimental impact on sleep deprivation compared to the *PER3* ^4/4^ genotype [34–37]. Subjects with the *PER3* ^5/5^ genotype showed good quality of sleep; however, under sleep deprivation conditions, demonstrated poor cognitive capacity [34–36, 39], declined time-on-task performance [37], and greater sleepiness [34]. It is possible that sleep deficiency-related vulnerability in attention may be enhanced by particular genotypes under sleep deficient conditions. These findings raise the possibility that the diminished morning gastric motility observed in the subjects with the longer 5-repeat allele (*PER3* ^4/5^ or *PER3* ^5/5^ genotypes) may be a physiological vulnerability in response to short sleep time. It has been reported that Japanese women’s sleep duration is the shortest in the world [40, 41]; participants of the present study were also short-time sleepers (approximately six hours, see Table 1, 2). Being in such a chronically vigilant state may selectively influence gut motility in the longer 5-repeat allele carriers.

In addition to short-sleep, Chellappa et al. [42, 43] provided intriguing evidence that the exposure to blue-enriched light may attenuate sleep-wake regulation in subjects with the *PER3* ^5/5^ genotype. In short, the *PER3* ^5/5^ genotype exhibited a more pronounced alert response to a two-hour exposure to blue light in the evening. In modern society, there are many opportunities to be exposed to blue-enriched light via smart phones, mobile computers, and large-screen televisions. We hypothesize that the combination of light sensitivity and vulnerability to short-sleep may have a serious effect on the biological circadian system, particularly in *PER3* ^5/5^ individuals. To what extent this polymorphism may influence gut function warrants future studies under controlled laboratory conditions.

The present study has some limitations. First, this study had selection bias; all subjects were female university students, although the homogeneity of the studied population was a strength of the study. Further studies including different populations should be performed. Second, the signals recorded by EGG correlated to some degree with gastric emptying, and hence with gastric motility, but these signals may not be exactly correlated to actual gastric motility; therefore, the data should be interpreted carefully. Finally, ghrelin, following the circadian rhythm, is known to act as a stimulant on gastric motility, but was not measured. Future experiment for understanding the diurnal variations of gastric motility, gut hormones and related biological parameters is needed. Notwithstanding, the present study provides the first evidence of the sole and combinatorial effect of the *CLOCK* 3111T/C SNP and *PER3* VNTR polymorphism on morning gastric motility.

In conclusion, our results suggest that minor polymorphic genotypes of the circadian rhythm genes *CLOCK* and *PER3* may be associated with poor morning gastric motility, and may also have a combinatorial effect. These findings may offer a new viewpoint on the role of circadian rhythm genes on the peripheral circadian system, including the time-keeping function of the gut.
